# Comments on ‘Genotypic diversity of the Helicobacter pylori vacA c region and its correlation with gastric disease outcomes’

**DOI:** 10.1099/jmm.0.002002

**Published:** 2025-04-07

**Authors:** Masoud Keikha

**Affiliations:** 1 Tropical and Communicable Diseases Research Center, Iranshahr University of Medical Sciences, Iranshahr, Iran; 2 Department of Medical Microbiology, School of Medicine, Iranshahr University of Medical Sciences, Iranshahr, Iran

**Keywords:** gastric cancer, *Helicobacter pylori*, pathogenesis, *vacA*

**Dear editor**

The recent study by Khadir *et al*. [1] titled ‘Genotypic diversity of the *Helicobacter pylori vacA* c region and its correlation with gastric disease outcomes’ provides valuable insights into the association between *H. pylori vacA* c-region genotypes and gastric disease outcomes in a Moroccan cohort. Notably, the study reports a significant association between the *vacA* c1 allele and an increased risk of gastric cancer (GC). However, several issues should be considered to contextualize these findings and guide future research.

## Limited functional characterization of *vacA* c-region

While the study identifies correlations between *vacA* c-region genotypes and disease outcomes, it does not delve into the functional implications of these genotypic variations. The *vacA* gene’s other regions, such as the signal (s), middle (m), intermediate (i) and deletion (d) regions, have been extensively studied for their roles in *H. pylori* pathogenicity [2]. However, the specific contribution of the c-region to the bacterium’s virulence mechanisms remains unclear. Functional studies are necessary to elucidate how variations in the *vacA* c-region influence *H. pylori* pathogenicity and interaction with host tissues. To address this gap, clustered regularly interspaced short palindromic repeats (CRISPR)/Cas-based gene editing could be employed to create isogenic *H. pylori* strains with specific *vacA* c-region variants. These strains could then be tested in cellular or animal models to assess their effects on host cell vacuolation, apoptosis and immune response modulation. Additionally, computational modelling of the *vacA* protein structure could help predict how c-region variations alter toxin activity or host receptor binding. For example, prior studies using CRISPR/Cas to investigate *H. pylori* virulence factors, such as *cagA*, have successfully elucidated functional mechanism [3].

## Potential confounding factors

The study does not account for other *H. pylori* virulence factors, such as the presence of the *cagA* gene, which have been associated with severe gastric diseases [4]. The interplay between multiple virulence factors may modulate the bacterium's overall pathogenic potential. Additionally, host genetic factors (e.g. polymorphisms in IL-1β or TNF-α genes) and environmental influences (e.g. diet, smoking or socioeconomic status), which were not considered in the study, could confound the observed associations. To integrate these confounders into future analyses, multivariate regression models could be employed to adjust for host and environmental variables. For instance, studies by El-Omar *et al*. [5] have demonstrated the importance of incorporating host genetic polymorphisms when assessing *H. pylori*-related disease risk. Environmental factors could be assessed using standardized questionnaires or biomarkers, as seen in studies linking high-salt diets to *H. pylori* virulence [6].

## Cross-sectional study design

The cross-sectional nature of the study limits the ability to infer causal relationships between *vacA* c-region genotypes and gastric disease outcomes. Longitudinal studies tracking individuals over time are necessary to establish temporal associations and causality. Such studies would provide stronger evidence for the role of specific *vacA* c-region genotypes in the progression from *H. pylori* infection to gastric pathology. Prospective cohort studies could be designed to follow individuals with *H. pylori* infection over decades, monitoring changes in *vacA* genotypes and disease outcomes. For example, the Shandong Intervention Trial successfully used a longitudinal design to demonstrate the long-term benefits of *H. pylori* eradication in reducing GC incidence [7]. Similarly, nested case-control studies within large cohorts could provide insights into the temporal dynamics of *vacA* c-region variations and disease progression.

## Methodological considerations

The detection of multiple infections, indicated by the presence of both *vacA* c1 and c2 alleles in some samples, suggests the possibility of mixed *H. pylori* populations within individual hosts [8]. This could complicate the interpretation of genotype–disease associations. Advanced molecular techniques, such as deep sequencing or single-cell genomics, should be employed in future studies to accurately characterize the genetic diversity of *H. pylori* within hosts and its clinical implications. For instance, deep sequencing has been successfully used in studies by Kennemann *et al*. [9] to resolve mixed *H. pylori* infections and track strain evolution within hosts. Single-cell genomics could further elucidate the heterogeneity of *H. pylori* populations and their functional consequences at the cellular level.

## Conclusion

While Khadir *et al*.’s study contributes to understanding the potential role of *vacA* c-region genotypes in gastric disease outcomes, addressing these limitations through further research is crucial. Future studies should aim for broader population inclusion, functional analyses of *vacA* c-region variants, consideration of additional bacterial and host factors, longitudinal designs and refined methodological approaches to validate and expand upon these findings. By integrating these suggestions, researchers can provide a more comprehensive understanding of the *vacA* c-region’s role in *H. pylori*-associated gastric diseases.

## Abbreviations

CRISPR: clustered regularly interspaced short palindromic repeats
GC: gastric cancer
*H. pylori*: *Helicobacter pylori*
